# Placebo Effects in the Context of Religious Beliefs and Practices: A Resting-State Functional Connectivity Study

**DOI:** 10.3389/fnbeh.2021.653359

**Published:** 2021-05-06

**Authors:** Anne Schienle, Andreas Gremsl, Albert Wabnegger

**Affiliations:** Department of Clinical Psychology, University of Graz, BioTechMed, Graz, Austria

**Keywords:** resting-state functional connectivity, religious belief, fronto-parietal cognitive control network, salience network, placebo

## Abstract

**Background**: Placebos (inert substances or procedures) can positively influence a person’s psychological and physical well-being, which is accompanied by specific changes in brain activity. There are many different types of placebos with different effects on health-related variables. This study investigated placebo effects in the context of religious beliefs and practices. The participants received an inert substance (tap water) along with the verbal suggestion that the water would come from the sanctuary in Lourdes (a major Catholic pilgrimage site with reports of miracle cures). We investigated changes in resting-state functional connectivity (rsFC) in three brain networks (default-mode, salience, cognitive control) associated with the drinking of the placebo water.

**Methods**: A total of 37 females with the belief that water from the sanctuary in Lourdes has positive effects on their spiritual, emotional, and physical well-being participated in this placebo study with two sessions. The participants drank tap water that was labeled “Lourdes water” (placebo) before a 15-min resting-state scan in one session. In the other (control) session, they received tap water labeled as tap water. The participants rated their affective state (valence, arousal) during the session and were interviewed concerning specific thoughts, feelings, and bodily sensations directly after each of the two sessions.

**Results**: The placebo reduced rsFC in the frontoparietal cognitive control network and increased rsFC in the salience network (insular-cerebellar connectivity). During the session, the participants rated their affective state as very pleasant and calm. The ratings did not differ between the two conditions. Immediately after the session, the participants reported increased intensity of pleasant bodily sensations (e.g., feelings of warmth, tingling) and feelings (e.g., gratefulness) for the “Lourdes water” condition.

**Conclusions**: The present findings provide the first evidence that placebos in the context of religious beliefs and practices can change the experience of emotional salience and cognitive control which is accompanied by connectivity changes in the associated brain networks.

## Introduction

A placebo is defined as “a substance or procedure … that is objectively without specific activity for the condition being treated” (Moerman and Jonas, 2002). The most common paradigm for assessing placebo effects (“placebo analgesia”) uses an inert intervention (e.g., a capsule filled with sugar, sham acupuncture, sham surgery) which is combined with the verbal suggestion of a pain-reducing treatment. Several studies have demonstrated that this approach leads to a reduction in experienced pain as well as altered brain activity in pain-sensitive regions and prefrontal cognitive control areas (for a summary see Wager and Atlas, 2015). Placebos have also been used in other areas. Placebo treatment can reduce the intensity of negative affective states (e.g., anxiety, disgust), which is accompanied by changes in brain activity in regions involved in the encoding of affective salience (e.g., insula, anterior cingulate cortex; Petrovic et al., 2005; Schienle et al., 2014). Moreover, placebos have been successfully administered to improve physical well-being and sports performance (Beedie and Foad, 2009), emotional and social well-being (e.g., interpersonal trust; Yan et al., 2018). Thus, research shows that there is not one single placebo effect, but many (Benedetti, 2014).

Another very important area of placebo application involves the treatment of illness. Placebo-induced symptom reduction has been reported for several diseases and mental disorders, such as Parkinson’s disease, depression, attention-deficit hyperactivity disorder, and binge-eating disorder (De la Fuente-Fernández and Stoessl, 2002; Weimer et al., 2015). Illness typically involves psychological aspects; patients not only sense somatic signs of illness, but they interpret these signs. Interpretations, such as cognitions of danger or loss (e.g., the threat of dying, loss of health) produce anxiety or depressed mood. A placebo counteracts these negative interpretations (Lundh, 1987). Patients who believe that a placebo is going to improve their health condition will experience reduced stress and anxiety; and these processes are accompanied by specific neurobiological processes (e.g., altered activation in cognitive control areas of the brain (Benedetti, 2014).

The present investigation focused on a specific placebo in the context of religious beliefs and practices. The participants received an inert substance (tap water), which was administered with the verbal suggestion that it is water from the sanctuary in Lourdes (a major Catholic pilgrimage site in France). Many Roman Catholics believe that Lourdes water has supernatural healing powers and the Medical Bureau of Lourdes has been recorded more than 7,000 reports of cured diseases^1^ (December, 9th, 2020).

Geochemical analyses of the water from different springs in the Lourdes area have shown that the water contains little total dissolved solids, has a slightly alkaline pH level (7.50–7.68), and oxidizing conditions, all of which are typical characteristics of a hydrogeological system that developed in carbonate-dominated bedrock (Dobrzyński and Rossi, 2017). There are no “special” ingredients in the water. Therefore, it does not seem to be the water in itself that has a positive effect, but the belief in it.

Placebo effects require that the treated person believes that a specific treatment or procedure will work. It has been shown that the expectancy and the desire for improvement are positively correlated with the magnitude of the placebo effect (e.g., Enck et al., 2008). Thus, what we believe we will experience from a treatment has a substantial impact on what we experience. Moreover, spirituality is associated with placebo responsivity. Hyland et al. (2006) showed that spirituality predicted perceived improvement of individual problems, such as unexplained fears and worries, after placebo (flower essence) treatment independently of expectancy.

In the present study, we focused on resting-state functional connectivity (rsFC), which is widely used in neuroscience research to investigate intrinsic neural circuits and their functional states (for a summary see Uddin et al., 2019). The term “resting state” refers to a state in which the individual is awake (lying quietly with eyes closed) and does not perform a specific experimental task (Raichle et al., 2001). Several large-scale functional brain networks have been identified during resting states, such as the default-mode network (involved in self-referential processing, mentalizing), the salience network (involved in detecting/integrating interoceptive, autonomic, and emotional information), and the frontoparietal cognitive control network (involved in the deliberate selection of thoughts, emotions, and behaviors; for a description of the networks see Marek and Dosenbach, 2018; Uddin et al., 2019). For example, it has been shown that religiously inspired techniques, such as mindfulness meditation can increase rsFC in the default-mode network between the posterior cingulate cortex and the dorsolateral prefrontal cortex (Creswell et al., 2016).

To the best of our knowledge, this is the first study to explore the effects of (Christian) religious belief on rsFC in different functional networks (cognitive control, salience, default-mode). We focused on these networks because previous neuroimaging research has indicated that placebo responding as well as religious/spiritual experiences are linked to the structure and function of neural components of these networks (e.g., prefrontal cortex, insula, superior/posterior parietal regions; e.g., Wiech et al., 2008; Schjoedt et al., 2009; McClintock et al., 2019; Schienle et al., 2019). The mentioned brain areas are involved in emotion regulation, attention control, and self-awareness (Tang et al., 2015). For example, in the study by Wiech et al. (2008), practicing Catholics and non-religious participants received noxious stimulation while they were either presented with an image of the Virgin Mary or a portrait without religious connotation. The religious group perceived less pain while looking at the religious image, which was associated with increased activation in the ventrolateral prefrontal cortex. This area plays a central role in pain modulation *via* reappraisal.

In the present study, females who believed in the miracles of Lourdes drank tap water directly before two resting-state scans separated by approximately 1 week. The water was labeled “water from the sanctuary of Lourdes” in one condition (placebo), and “tap water” in another condition (control). It was tested, whether the placebo would change reported well-being (emotional, cognitive, bodily) and rsFC in brain networks (cognitive control, salience, default-mode) compared to the control condition.

## Materials and Methods

### Sample

A total of 37 females (mean age: *M* = 30.59 years, SD = 13.8) participated in this study. To reduce sex-related variance in resting-state functional connectivity (e.g., Weis et al., 2020), and religious well-being (Unterrainer et al., 2010) the sample was restricted to females. They had been selected based on their answers in an internet-based survey that contained the following questions: (a) Do you think that water from the sanctuary in Lourdes can have positive (spiritual, emotional, somatic) effects?; (b) Would you use Lourdes water if you had a serious illness?; and (c) Do you believe in miracles in a religious/spiritual sense? The questions were answered on Likert scales ranging from 0 = “no” to 6 = “definitely.” The inclusion criterion for the study was an average “Lourdes score” >3 (*M* = 3.76, SD = 1.1). The Lourdes score had sufficient reliability (McDonald’s omega = 0.75).

All of the participants had a highschool diploma; 76% were University students and the remaining participants were white-collar workers. The religious affiliation of the majority of participants was Roman–Catholic (73%), while others stated to be Protestants (6%), or not religiously affiliated (21%). None of the participants reported a current serious somatic illness (e.g., cancer, neurological disease) or mental disorder.

### Stimuli and Design

The study had a repeated-measures design. The participants were scanned twice and received a glass of tap water (60 ml) labeled as water from the sanctuary in Lourdes (placebo) in one session and tap water labeled as tap water in the other session (control). The sequence of the sessions was counterbalanced.

### Procedure

We invited the participants to two functional magnetic resonance imaging (fMRI) sessions, which were scheduled 1 week apart from each other. In the placebo condition, the participants first obtained written information (one sheet) describing the religious visions of Saint Bernadette of Lourdes. In the control condition, the sheet provided basic knowledge about fMRI. The information in the two conditions was also summarized as a poster on the wall of the room, where the instruction took place. Subsequently (directly before the MRI recording), the water was served. The water was poured out of a little bottle into the glass. The label of the bottle either stated “Sanctuary of Lourdes” or “tap water.”

The fMRI session started with the resting-state measurement. The experimenter who conducted the scanning was not aware of the condition. The participants were instructed “Close your eyes and let your thoughts wander freely.” Directly after the 15-min resting-state scan, the participants rated their experience on two basic affective dimensions, valence, and arousal, on 9-point Likert scales (9 = very pleasant, aroused). Afterward, a structural scan was obtained (duration: 4.5 min). Following the MRI session, the participants were asked by the experimenter to report specific thoughts, feelings, and somatic sensations experienced during the MRI recording. Each reported symptom was rated on a 9-point Likert scale according to experienced intensity (9 = very intense). The participants also rated the “overall effects of the water” on a 9-point scale (9 = very strong) and were invited to give open comments.

Finally, the participants completed the scale “general religiosity” of the multidimensional instrument for the measurement of religious-spiritual well-being (MI-RSWB 48 by Unterrainer et al., 2010). The scale contains eight statements (e.g., My faith gives me a feeling of security; Cronbach’s alpha = 0.94), which are answered on a 6-point Likert scale ranging from 1 = “strongly disagree” to 6 = “strongly agree.”

All participants received detailed instructions before data collection. The study protocol was approved by the ethics committee of the University (GZ: 39/19/63 ex 2018/19). Following their study involvement, all participants were fully debriefed and received written information about the aim of the study, the procedure (placebo approach), and the main findings of the experiment. We also offered the option of personal communication with the experimenter.

### MRI Recording and Analysis

The MRI session was conducted with a 3T scanner (Skyra, Siemens, Erlangen, Germany) with a 32-channel head coil. In both sessions (“Lourdes water”, Tap water) structural images were recorded using a T1-weighted MPRAGE sequence with following settings: TR = 1.680 s; TE = 0.00188 s; acquisition matrix = 256; flip angle = 8°; 192 transverse slices; FoV = 224 mm; slice thickness = 0.88 mm; fat suppression: water excit. fast; acquisition time = 4.29 s). Functional images were acquired using a CMRR-multiband^2^ with following settings: sequence type = epfid, acceleration factor = 3; TR = 1.4 s; TE = 0.0304 s; flip angle = 72 degrees; slice thickness = 3 mm; total readout time = 0.046 s; spacing between slices: 3 mm; acquisition matrix = 80; phase encoding direction: anterior-posterior; number of volumes = 650).

Resting-state analyses were carried out by using the CONN toolbox (version 18.b^3^, RRID:SCR_009550; Whitfield-Gabrieli and Nieto-Castanon, 2012) and SPM12^4^ (version 7487) implemented in Matlab (2017b). Preprocessing followed the default pipeline suggested by the CONN toolbox (realignment, slice-timing, normalization, and spatial smoothing with an 8 mm Gaussian kernel to ameliorate individual anatomical differences). The final voxel size was 3 mm isotropic. The subsequent component-based noise-reduction approach (CompCor) included 15 dimensions of white matter and cerebrospinal fluid, as well as 12 realignment parameters (including 1st-order derivatives). Scrubbing controlled for additional motion-related variance. Finally, a bandpass filter (0.0–10.1 Hz) was applied.

### Statistical Analysis

#### Self-report

We used paired *t*-tests to compare the effects of Condition (PLACEBO: “Lourdes water” vs. CONTROL: Tap water) on the ratings reported in the scanner (arousal, pleasure) and outside of the scanner (intensity of experienced emotions, thoughts, bodily sensations, perceived effectiveness of the treatment). Additionally, we computed Pearson correlations between questionnaire scores (general religiosity, the perceived overall effect of the “Lourdes water”). We used the Bonferroni-Holm correction for multiple comparisons (Holm, 1979).

#### fMRI Data

We computed weighted GLM bivariate region of interest (ROI) correlations between 29 network ROIs provided by the CONN toolbox. The labeling of each ROI can be found in the supplementary material. The ROIs are based on the analysis of the human connectome project (HCP) dataset with 497 subjects. In the second-level analysis step, we investigated the contrast PLACEBO—CONTROL. Age was introduced as a covariate because of the substantial age range (18–58 years). Moreover, we computed exploratory correlation analyses between resting-state connectivity and difference scores of self-reports (e.g., the intensity of bodily sensations in the placebo condition minus bodily sensations in the control condition). We used false-discovery rate (FDR) seed-level correction provided by the CONN-toolbox, that corrects across multiple target areas. Results were considered statistically significant if *p*_(FDR)_ < 0.05.

## Results

### Self-reports

#### Ratings in the Scanner

The conditions (PLACEBO: “Lourdes water” vs. CONTROL: Tap water) did not differ concerning reported valence and arousal (see Table 1).The participants experienced their affective state as very pleasant (*M* = 6.56, SD = 1.36) and calm (*M* = 2.63, SD = 1.45) across both conditions.

**Table 1:** Self-reports for the conditions with “Lourdes water” suggestion vs. “tap water” suggestion.

	PLACEBO: “Lourdes water”	CONTROL: “tap water”	T_36_ (p)	Cohen’s *d*
	M (SD) [95% BCa CI]	M (SD) [95% BCa CI]		
	Inside-scanner ratings (1‥9)			
Pleasantness	6.81 (1.57) [6.24–7.32]	6.31 (1.86) [5.62–6.96]	1.42 (0.165)	0.23
Arousal	2.54 (1.71) [2.06–3.11]	2.72 (1.87) [2.19–3.28]	0.51 (0.617)	0.08
	Outside-scanner ratings (intensity 1‥9)			
Thoughts	5.09 (2.11) [4.29–5.76]	4.89 (1.97) [4.26–5.57]	0.53 (0.599)	0.09
Feelings	6.15 (1.96) [5.28–6.76]	4.99 (2.16) [4.17–5.92]	2.90 (0.006)	0.48
Bodily sensations	4.47 (2.19) [3.59–5.27]	3.30 (1.58) [2.37–3.84]	2.58 (0.014)	0.42
Estimated overall effect of the water	4.35 (2.38) [3.62–5.09]	1.89 (1.33) [1.46–2.44]	6.60 (0.001)	1.09

#### Ratings Outside of the Scanner

The participants rated the water as more effective in the placebo condition than in the control condition (*p* = 0.001). The perceived intensity of bodily sensations and feelings was higher after the application of “Lourdes water” compared to tap water labeled as such (*p*s < 0.015). The intensity of thoughts did not differ between the conditions (Table 1). The reported bodily sensations involved skin sensations (tingling), feeling of warmth, and “bodily relaxation” (mentioned by *n* = 25 participants; 68%). Thoughts mainly involved other people (friends, partners), daily duties, and past experiences (e.g., movies, parties). Specific emotions that were reported included happiness, satisfaction, gratefulness, anxiety, and nervousness. Negative emotions were predominantly reported in the control condition (CONTROL vs. PLACEBO; nervousness: 8 vs.1; anxiety: 5 vs. 0).

#### Open Comments

Of the participants, 32% (*n* = 12) reported a specific taste of the “Lourdes water” (e.g., “tastes like spring water”, “tastes fresher than tap water”). Twelve participants stated that they experienced a different time perception in the “Lourdes water” condition compared to the tap water condition (“Time went by faster”). One participant reported a considerable symptom reduction concerning her chronic obstructive pulmonary disease after the “Lourdes water” condition (reduced breathing problems). Nine participants (24%) reported a religious experience during the “Lourdes water” condition, such as mental images of Jesus at the cross, the grotto of Lourdes, or Saint Bernadette.

#### Exploratory Correlation Analyses

The mean score on the general religiosity scale (MI-RSWB 48 by Unterrainer et al., 2010) was *M* = 3.65 (SD = 1.40). This score was positively correlated with the “overall effect” of the “Lourdes water” (*r* = 0.524, *p* = 0.001). Additionally, the “Lourdes score” (belief in the healing power of Lourdes water) was positively correlated with “general religiosity” *(r* = 0.496, *p* = 0.002), the intensity of reported feelings in the “Lourdes water” condition (*r* = 0.425, *p* = 0.009), and with the estimated “overall effect” of the “Lourdes water” (*r* = 0.354, *p* = 0.031).

### Resting-State Functional Connectivity

**Figure 1:**
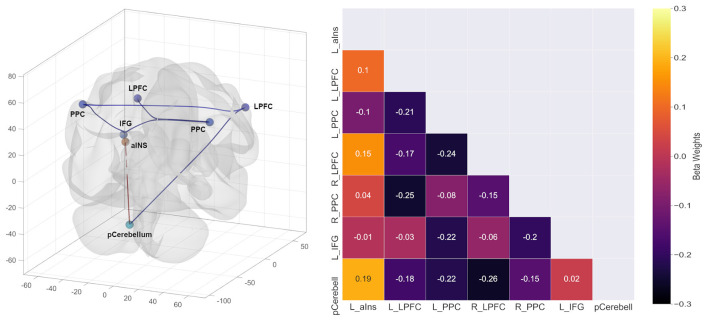
Results of the resting-state functional connectivity analysis.

The findings of the rsFC analysis are displayed in Table 2 and Figure 1. The placebo condition (compared to the control condition) was associated with reduced rsFC between the posterior parietal cortex (PPC) and the lateral prefrontal cortex (LPFC) as well as the inferior frontal gyrus (IFG). Moreover, reduced rsFC was observed between the cerebellum and the LPFC.

Increased rsFC during the “Lourdes water” condition (compared to control) characterized the anterior insula and the cerebellum.

**Table 2:** Results of the resting-state functional connectivity analysis.

ROI (seed)	ROI	*t*	*p*(FDR)
**CONTROL (“Tap water”)–PLACEBO (“Lourdes water”)**
Posterior parietal cortex (l)	Inferior frontal gyrus (l)	−4.28	0.004
Posterior parietal cortex (l)	Lateral prefrontal cortex (r)	−3.50	0.018
Posterior parietal cortex (r)	Inferior frontal gyrus (l)	−3.27	0.049
Posterior parietal cortex (r)	Lateral prefrontal cortex (l)	−3.13	0.049
Lateral prefrontal cortex (r)	Posterior parietal cortex (l)	−3.50	0.037
Lateral prefrontal cortex (r)	Posterior cerebellum	−3.24	0.037
Inferior frontal gyrus (l)	Posterior parietal cortex (l)	−4.28	0.004
Inferior frontal gyrus (l)	Posterior parietal cortex (r)	−3.27	0.034
Posterior cerebellum (r)	Lateral prefrontal cortex (r)	−3.24	0.044
**PLACEBO (“Lourdes water”)–CONTROL (“Tap water”)**
Posterior cerebellum (r)	Anterior insula (l)	3.17	0.044

*ROI, region of interest; l/r: left/right; FDR, false discovery rate*.

#### Exploratory Analyses

To follow up on the finding that “Lourdes water” increased the intensity of pleasant bodily sensations and feelings, we correlated rsFC with the difference scores for the ratings in the two conditions (PLACEBO minus CONTROL). Changes in bodily sensations were positively correlated with rsFC between occipital and inferior frontal regions, and negatively with rsFC between inferior frontal regions and temporal/parietal regions (Table 3). Changes in the intensity of feelings were not correlated with rsFC.

**Table 3:** Association between resting-state functional connectivity and experienced bodily changes (“PLACEBO: Lourdes water”–“CONTROL: Tap water”).

ROI (seed)	ROI	*t*	*p*(FDR)
Occipital cortex (l)	Inferior frontal gyrus (l)	3.79	0.017
Inferior frontal gyrus (l)	Occipital cortex (l)	3.79	0.015
Superior temporal gyrus (r)	Inferior frontal gyrus (r)	−3.77	0.015
Superior temporal gyrus (r)	Inferior frontal gyrus (l)	−3.59	0.015
Superior temporal gyrus (l)	Supramarginal gyrus (r)	−3.48	0.039
Supramarginal gyrus (r)	Superior temporal gyrus posterior (l)	−3.48	0.039
Inferior frontal gyrus (r)	Inferior frontal gyrus (l)	−3.59	0.015
Inferior frontal gyrus (r)	Superior temporal gyrus posterior (r)	−3.77	0.017
Inferior frontal gyrus (l)	Superior temporal gyrus posterior (r)	−3.59	0.015

*Note: experienced bodily changes: intensity of bodily sensation in the Lourdes water condition minus intensity of bodily sensation in the Tap water condition; ROI, region of interest; l/r, (left, right); negative/positive t-score indicates the direction of correlation; FDR, false discovery rate*.

Addionally, we compared participants with Roman-Catholic affilitation (*n* = 27) and other affiliations (*n* = 10) with each other concerning resting-state connectivity and questionnaire/rating data. Roman-Catholics reported greater general religiosity on the MI-RSWB 48 (Unterrainer et al., 2010; *M* = 3.94, SD = 1.32) compared to participants who were not Roman-Catholic (*M* = 2.88, SD = 1.40; *t*_(35)_ = 2.15, *p* = 0.039). All other variables (ratings and resting-state) did not reveal statististically significant group differences (*p* > 0.05).

## Discussion

This resting-state functional connectivity (rsFC) study examined a specific placebo. The participants received an inert substance (tap water) along with the verbal suggestion that they would drink water from the sanctuary in Lourdes. Compared to drinking tap water labeled as such, “Lourdes water” changed the strength of temporal correlations between specific brain sites, including both increased as well as decreased connectivity.

The placebo increased the connectivity between the anterior insula and the posterior cerebellum. Both regions are part of the salience network (e.g., Habas et al., 2009; Uddin, 2015). The salience network is involved in detecting, integrating, and filtering interoceptive, autonomic, and emotional information. The label “salience” is applied to this network for its broad role in identifying (subjectively) important, or salient, information. It has been shown that the salience network with the insula as a central hub mediates placebo effects in different areas (e.g., placebo analgesia, reduction of negative affect; Schienle et al., 2014; Wager and Atlas, 2015). The insular cortex can integrate and transform information about salience into perceptual decisions. This brain region links emotional/motivational/decision processes, which is central for placebo responding (Wager and Atlas, 2015). Moreover, the role of the insula in spiritual experience has been identified before (Hölzel et al., 2008; Haase et al., 2016). An fMRI study by Haase et al. (2016) showed that religiously inspired training (20-h mindfulness training) altered insula activation to a stressor (loaded breathing).

In line with the observed changes in rsFC within the salience network (insula-cerebellum connectivity), the “Lourdes water” application increased the intensity of experienced positive feelings (e.g., gratefulness) and bodily sensations. Many participants experienced tingling and warming, which they interpreted as signs of “bodily relaxation.” On the one hand, it is well-known that tingling and warming are associated with autonomic relaxation (i.e., an increase in parasympathetic activity and a decrease in sympathetic activity). These sensations are typically experienced by individuals who practice relaxation training. On the other hand, focusing one’s attention on a body part can give rise to various “spontaneous sensations” without external stimulation. These attention-related sensations strongly depend on expectations and prior information (Tihanyi et al., 2018). In the present study, the “Lourdes water” suggestion implied possible somatic changes since water from the sanctuary has been linked with the healing of somatic illness.

It is noteworthy, that the placebo-induced change in the bodily state was correlated with frontoparietal rsFC. The connectivity within this network was generally reduced through the placebo. More specifically, the coupling of the IFG and the lateral prefrontal cortex (LPFC) with the PPC was lowered. The mentioned regions are part of a frontoparietal cognitive control network (e.g., Dosenbach et al., 2008; Dixon et al., 2018; Marek and Dosenbach, 2018). Cognitive control refers to the deliberate selection of thoughts, emotions, and behaviors based on current task demands and social context, as well as inhibition of inappropriate actions (Miller and Cohen, 2001). An influential model suggests that the lateral prefrontal cortex (LPFC) represents rules or instructions in working memory. This information guides perceptual and motor processing in parietal regions, thus resulting in action selection or inhibition (Miller and Cohen, 2001). The LPFC represents relationships between contexts, task rules, and anticipated outcomes (Dixon et al., 2018). The mentioned functions are central for placebo responding, which typically involves the LPFC (e.g., Wager et al., 2004; Petrovic et al., 2010). Placebos only work if the recipients believe in the effectiveness of the treatment. The belief is associated with anticipation and positive outcome expectations, which are represented in the LPFC.

Functional brain imaging studies with a localization approach have detected associations between neural activation in lateral prefrontal regions (Wiech et al., 2008), the IFG (Kapogiannis et al., 2009), superior/posterior parietal regions (Kapogiannis et al., 2009), and religious/spiritual experiences. In the study by Kapogiannis et al. (2009), the participants indicated whether they agreed to religious statements (e.g., addressing God’s involvement in the world) or not. In a reanalysis of the data set, the authors (Kapogiannis et al., 2014) focused on effective connectivity (causal binding) between specific brain regions. They identified a pathway from the IFG to the superior medial frontal gyrus, and the precuneus when the participants were thinking about God’s level of involvement. Thus, the placebo intervention of the present investigation affected the connectivity of those brain areas involved in both, placebo responding and spiritual experience.

This study has several limitations that merit consideration. First, we only studied females. Therefore, the results cannot be generalized to males. Second, the time for recording rsFC was relatively short. The reliability of resting-state correlations can be increased with longer periods of data acquisition (~45 min) (Marek and Dosenbach, 2018). Third, we investigated a sample of healthy individuals. The placebo effects on rsFC can be possibly enhanced by studying a sample with a greater need for positive effects of “Lourdes water” (e.g., patients with various illnesses). However, this approach has ethical issues.

The present study differs from previous placebo research regarding the use of verbal suggestions. Typical instructions in placebo studies involve a clear statement of the expected effect (e.g., “this pill will reduce your pain”). In contrast, in the present investigation, each participant had to create her own “instruction” based on her concept about Lourdes water effects. Some authors have pointed out that when individuals report their experience through concepts and beliefs, they significantly distort their direct experience (Pashko, 2013). They report opinions instead of direct experience. This might also explain, why rating differences between the placebo and control conditions were more pronounced when interpreting the experience after the session instead of during the session.

In summary, the findings of the present study allow us to draw preliminary conclusions about the placebo effect in the context of religious beliefs and practices. We found that this type of placebo can enhance emotional-somatic well-being, and can lead to changes in rsFC in cognitive control/emotional salience networks of the brain. Future research is warranted to replicate the results. Moreover, future research should investigate whether the observed effects generalize across different religious affiliations. The idea of “holy water” (or blessed water) is common in several religions, from Christianity, Islam, Buddhism to Sikhism.

## Data Availability Statement

The datasets generated during and/or analyzed during the current study are available from the corresponding author on reasonable request.

## Ethics Statement

The studies involving human participants were reviewed and approved by ethics committee of the University (GZ: 39/19/63 ex 2018/19). The patients/participants provided their written informed consent to participate in this study.

## Author Contributions

All authors were involved in the conception of this study, data analysis and writing of the manuscript. All authors contributed to the article and approved the submitted version.

## Conflict of Interest

The authors declare that the research was conducted in the absence of any commercial or financial relationships that could be construed as a potential conflict of interest.

## Footnotes

^1^https://www.lourdes-france.org/en/miraculous-healings/retrieved

^2^https://www.cmrr.umn.edu/multiband/

^3^www.nitrc.org/projects/conn

^4^https://www.fil.ion.ucl.ac.uk/spm/software/spm12/

## References

[B1] Beedie J. Foad A. J. (2009). The placebo effect in sports performance. A brief review. Sports Med. 39, 313–329. 10.2165/00007256-200939040-00004 19317519

[B2] Benedetti F. (2014). Placebo effects: from the neurobiological paradigm to translational implications. Neuron 84, 623–637. 10.1016/j.neuron.2014.10.023 25442940

[B3] Creswell J. D. Taren A. A. Lindsay E. K. Greco C. M. Gianaros P. J. Fairgrieve A.. (2016). Alterations in resting-state functional connectivity link mindfulness meditation with reduced interleukin-6: a randomized controlled trial. Biol. Psychiatry 80, 53–61. 10.1016/j.biopsych.2016.01.008 27021514

[B4] De la Fuente-Fernández R. Stoessl A. J. (2002). The placebo effect in Parkinson’s disease. Trends Neurosci. 25, 302–306. 10.1016/s0166-2236(02)02181-1 12086748

[B5] Dixon M. L. De La Vega A. Mills C. Andrews-Hanna J. Spreng R. N. Cole M. W.. (2018). Heterogeneity within the frontoparietal control network and its relationship to the default and dorsal attention networks. Proc. Natl. Acad. Sci. U S A 115, E1598–E1607. 10.1073/pnas.1715766115 29382744 PMC5816169

[B6] Dobrzyński D. Rossi D. (2017). Geochemistry of trace elements in spring waters of the Lourdes area (France). Annales Societatis Geologorum Poloniae 87, 199–212. 10.14241/asgp.2017.010

[B7] Dosenbach N. U. Fair D. A. Cohen A. L. Schlaggar B. L. Petersen S. E. (2008). A dual-networks architecture of top-down control. Trends Cogn. Sci. 12, 99–105. 10.1016/j.tics.2008.01.001 18262825 PMC3632449

[B9] Enck P. Benedetti F. Schedlowski M. (2008). New insights into the placebo and nocebo responses. Neuron 59, 195–206. 10.1016/j.neuron.2008.06.030 18667148

[B10] Haase L. Thom N. J. Shukla A. Davenport P. W. Simmons E. N. Stanley E. A.. (2016). Mindfulness-based training attenuates insula response to an aversive interoceptive challenge. Soc. Cogn. Affect. Neurosci. 11, 182–190. 10.1093/scan/nsu042 24714209 PMC4692309

[B11] Habas C. Kamdar N. Nguyen D. Prater K. Beckmann C. F. Menon V.. (2009). Cerebellar contributions to intrinsic connectivity networks. J. Neurosci. 29, 8586–8594. 10.1523/JNEUROSCI.1868-09.2009 19571149 PMC2742620

[B12] Holm S. (1979). A simple sequential rejective multiple test procedure. Scand. J. Stat. 6, 65–70. 10.2307/4615733

[B13] Hölzel B. K. Ott U. Gard T. Hempel H. Weygandt M. Morgen K.. (2008). Investigation of mindfulness meditation practitioners with voxel-based morphometry. Soc. Cogn. Affect. Neurosci. 3, 55–61. 10.1093/scan/nsm038 19015095 PMC2569815

[B14] Hyland M. E. Geraghty A. Joy O. Turner S. (2006). Spirituality predicts outcome independently of expectancy following flower essence self-treatment. J. Psychosom. Res. 60, 53–58. 10.1016/j.jpsychores.2005.06.073 16380310

[B15] Kapogiannis D. Barbey A. K. Su M. Zamboni G. Krueger F. Grafman J.. (2009). Cognitive and neural foundations of religious belief. Proc. Natl. Acad. Sci. U S A 106, 4876–4881. 10.1073/pnas.0811717106 19273839 PMC2660736

[B16] Kapogiannis D. Deshpande G. Krueger F. Thornburg M. P. Grafman J. H. (2014). Brain networks shaping religious belief. Brain Connectivity 4, 70–79. 10.1089/brain.2013.0172 24279687 PMC3929007

[B18] Lundh S. G. (1987). Placebo, belief and health. A cognitive-emotion model. Scand. J. Psychol. 28, 128–143 10.1111/j.1467-9450.1987.tb00747.x 3317812

[B19] Marek S. Dosenbach N. U. (2018). The frontoparietal network: function, electrophysiology and importance of individual precision mapping. Dialogues Clin. Neurosci. 20, 133–140. 10.31887/DCNS.2018.20.2/smarek 30250390 PMC6136121

[B20] McClintock C. H. Worhunsky P. D. Xu J. Balodis I. M. Sinha R. Miller L.. (2019). Spiritual experiences are related to engagement of a ventral frontotemporal functional brain network: implications for prevention and treatment of behavioral and substance addictions. J. Behav. Addict. 8, 678–691. 10.1556/2006.8.2019.71 31891313 PMC7044576

[B21] Miller E. K. Cohen J. D. (2001). An integrative theory of prefrontal cortex function. Annu. Rev. Neurosci. 24, 167–202. 10.1146/annurev.neuro.24.1.167 11283309

[B23] Moerman D. E. Jonas W. B. (2002). Deconstructing the placebo effect and finding the meaning response. Anna. Intern. Med. 136, 471–476. 10.7326/0003-4819-136-6-200203190-00011 11900500

[B24] Pashko W. (2013). Shifting between our two self-identities can cause the placebo effect and reponse shift. J. Transpersonal Psychol. 45, 8–23. 10.1177/002216787701700308

[B25] Petrovic P. Dietrich T. Fransson P. Andersson J. Carlsson K. Ingvar M.. (2005). Placebo in emotional processing—induced expectations of anxiety relief activate a generalized modulatory network. Neuron 46, 957–969. 10.1016/j.neuron.2005.05.023 15953423

[B26] Petrovic P. Kalso E. Petersson K. M. Andersson J. Fransson P. Ingvar M.. (2010). A prefrontal non-opioid mechanism in placebo analgesia. Pain 150, 59–65 10.1016/j.pain.2010.03.011 20399560

[B27] Raichle M. E. MacLeod A. M. Snyder A. Z. Powers W. J. Gusnard D. A. Shulman G. L.. (2001). A default mode of brain function. Proc. Natl. Acad. Sci. U S A 98, 676–682. 10.1073/pnas.98.2.676 11209064 PMC14647

[B28] Schienle A. Höfler C. Wabnegger A. (2019). Belief in the miracles of Lourdes: A voxel-based morphometry study. Brain Behav. 10:e01481. 10.1002/brb3.1481 31860792 PMC6955922

[B29] Schienle A. Übel S. Schöngaßner F. Ille R. Scharmüller W. (2014). Disgust regulation via placebo: an fMRI study. Soc. Cogn. Affect. Neurosci. 9, 985–990. 10.1093/scan/nst072 23868896 PMC4090961

[B30] Schjoedt U. Stødkilde-Jørgensen H. Geertz A. W. Roepstorff A. (2009). Highly religious participants recruit areas of social cognition in personal prayer. Soc. Cogn. Affect. Neurosci. 4, 199–207. 10.1093/scan/nsn050 19246473 PMC2686228

[B33] Tang Y. Y. Holzel B. K. Posner M. I. (2015). The neuroscience of mindfulness meditation. Nat. Rev. Neurosci. 16, 213–225. 10.1038/nrn3916 25783612

[B34] Tihanyi B. T. Ferentzi E. Beissner F. Köteles F. (2018). The neuropsychophysiology of tingling. Conscious. Cogn. 58, 97–110. 10.1016/j.concog.2017.10.015 29096941

[B35] Uddin L. Q. (2015). Salience processing and insular cortical function and dysfunction. Nat. Rev. Neurosci. 16, 55–61. 10.1038/nrn3857 25406711

[B36] Uddin L. Q. Yeo B. T. Spreng R. N. (2019). Towards a universal taxonomy of macro-scale functional human brain networks. Brain Topogr. 32, 926–942. 10.1007/s10548-019-00744-6 31707621 PMC7325607

[B37] Unterrainer H. F. Huber H. P. Ladenhauf K. H. Wallner-Liebmann S. J. Liebmann P. M. (2010). MI-RSB 48 Die entwicklung eines multidimensionalen inventars zum religiös-spirituellen befinden. Diagnostica 2, 82–93. 10.1026/0012-1924/a000001

[B38] Wager T. D. Atlas L. Y. (2015). The neuroscience of placebo effects: Connecting context, learning and health. Nat. Rev. Neurosci. 16, 403–418. 10.1038/nrn3976 26087681 PMC6013051

[B32] Wager T. D. Rilling J. K. Smith E. E. Sokolik A. Casey K. L. Davidson R. J.. (2004). Cohen Placebo-induced changes in fMRI in the anticipation and experience of pain. Science 303, 1162–1167 10.1126/science.1093065 14976306

[B39] Weimer K. Colloca L. Enck P. (2015). Placebo effects in psychiatry: mediators and moderators. The Lancet Psychiatry 2, 246–257. 10.1016/S2215-0366(14)00092-3 25815249 PMC4370177

[B40] Weis S. Patil K. R. Hoffstaedter F. Nostro A. Yeo B. T. Eickhoff S. B.. (2020). Sex classification by resting-state brain connectivity. Cereb. Cortex 30, 824–835. 10.1093/cercor/bhz129 31251328 PMC7444737

[B41] Whitfield-Gabrieli S. Nieto-Castanon A. (2012). Conn: A functional connectivity toolbox for correlated and anticorrelated brain networks. Brain Connectivity 2, 125–141. 10.1089/brain.2012.0073 22642651

[B42] Wiech K. Farias M. Kahane G. Shackel N. Tiede W. Tracey I.. (2008). An fMRI study measuring analgesia enhanced by religion as a belief system. Pain 139, 467–476. 10.1016/j.pain.2008.07.030 18774224

[B43] Yan X. Yong X. Huang W. Ma Y. (2018). Placebo treatment facilitates social trust and approach behavior. Proc. Natl. Acad. Sci. U S A 115, 5732–5737. 10.1073/pnas.1800779115 29760054 PMC5984523

